# Healthy living in pregnancy: a cluster-randomized controlled trial to prevent excessive gestational weight gain - rationale and design of the GeliS study

**DOI:** 10.1186/1471-2393-14-119

**Published:** 2014-03-28

**Authors:** Kathrin Rauh, Julia Kunath, Eva Rosenfeld, Luzia Kick, Kurt Ulm, Hans Hauner

**Affiliations:** 1 ZIEL – Research Centre for Nutrition and Food Sciences, Technische Universität München, Freising-Weihenstephan, Germany; 2 Competence Centre for Nutrition (KErn), Freising, Germany; 3 Else Kröner-Fresenius-Centre for Nutritional Medicine, Klinikum rechts der Isar, Uptown München Campus D, Technische Universität München, Munich, Germany; 4 Department of Medical Statistics and Epidemiology, Technische Universität München, Munich, Germany

**Keywords:** Lifestyle intervention, Gestational weight gain (GWG), Diet, Exercise, Gestational diabetes, Weight retention, Childhood obesity, Obesity prevention, Pregnancy

## Abstract

**Background:**

Recent studies suggest that excessive gestational weight gain (GWG) leads to adverse maternal and fetal outcomes including weight retention in the mother and an increased risk of childhood obesity in the offspring.

The aim of the GeliS study is to examine the effect of a lifestyle intervention programme during pregnancy to avoid excessive GWG and, hence, to reduce pregnancy and obstetric complications as well as the risk of maternal and offspring obesity.

**Methods and design:**

The GeliS study is a multicentre cluster-randomized controlled trial. A total number of 2500 pregnant women (singleton pregnancy) with a pre-pregnancy BMI ≥ 18.5 kg/m^2^ and ≤ 40 kg/m^2^ will be recruited in practices of gynaecologists and midwives in ten Bavarian regions. The intervention comprises three structured and individualised counselling sessions on a healthy diet, regular physical activity as well as weight monitoring during pregnancy and one session after delivery, respectively. The counselling sessions are attached to routine pre- and postnatal visits using standardised materials and procedures. In the control regions, general recommendations for a healthy lifestyle are given. An oral glucose tolerance test is offered to all participants.

The primary outcome is the proportion of participants with excessive GWG. Secondary outcomes include pregnancy and obstetric complications such as frequency of gestational diabetes, preeclampsia and caesarean sections as well as weight retention in the mothers and BMI and other health variables in the offspring. A 5-year follow-up of both mothers and their infants is planned.

**Discussion:**

The GeliS lifestyle intervention programme has been adapted to the existing routine health care system for pregnant women. If shown to be effective, it could be immediately implemented in routine care.

**Trial registration:**

The study protocol is registered at the ClinicalTrials.gov Protocol Registration System (NCT01958307).

## Background

The global prevalence of overweight and obesity has reached an epidemic dimension. In this context, the prevalence of overweight and obesity among women of reproductive age increased substantially [1,2]. Maternal overweight or obesity is associated with a variety of pregnancy and obstetric complications [3-7], such as gestational diabetes mellitus (GDM) [8], preeclampsia [9-11] and an increased risk for macrosomia [12] and caesarean delivery [13,14].

Likewise, recent data suggest that excessive GWG may result in a similar risk profile. Excessive GWG was found to be associated with an increased risk for many adverse maternal and fetal outcomes [15,16] such as large for gestational age (LGA) newborns [17-19] and long-term maternal weight retention after delivery [20-23]. Furthermore, GWG has been reported to be a significant risk factor for childhood obesity [24-33].

To support an optimal pregnancy outcome, the US Institute of Medicine (IOM) provided recommendations for GWG stratified according to BMI of the women before pregnancy [16]. Unfortunately, in many countries including the USA, but also in Germany, an increasing proportion of pregnant women exceed these recommended thresholds [16,25,34,35].

Growing evidence suggests that lifestyle during pregnancy and in the early postnatal period is considerably influencing fetal and infant growth and development. This is an important observation, as pregnancy and the early postnatal period represent critical stages of development for the offspring and may “programme” the risk of metabolic disorders later in life [36,37]. To interfere with this potentially vicious cycle [38,39], adequate intervention strategies which can be introduced into medical practice are urgently required. Some recent studies focussing on a healthy lifestyle during pregnancy and consisting of a balanced diet and regular physical activity showed a reduction in the risk of excessive GWG [40-43].

We recently performed the FeLIPO study (acronym for “Feasibility of a lifestyle intervention in pregnancy to optimize maternal weight development”) to assess the potential to prevent excessive GWG by a simple lifestyle intervention [44]. The 2-session intervention programme focussing on a healthy diet, regular physical activity, and self-monitoring of body weight resulted in a significantly lower proportion of women exceeding the IOM recommendations among women in the intervention group (38%) compared to the control group (60%, p < 0.05) [44]. Similar to the results of a recent meta-analysis [45], the FeLIPO study detected a trend for a decreased risk of developing gestational diabetes in the intervention group [44].

Based on the results of this pilot study we are planning to examine the potential of a comprehensive lifestyle programme in pregnant women under “real life” conditions. The GeliS project (acronym for “Gesund leben in der Schwangerschaft”/healthy living in pregnancy) is a public health approach focussing on the prevention of excessive GWG by lifestyle counselling on diet and physical activity during pregnancy, and by self-monitoring GWG using weight gain charts. The lifestyle intervention programme has been adapted to the German health care system to allow immediate implementation if it is shown to be effective at reducing the rate of excessive GWG and associated risks.

## Methods and design

### Study design and setting

The GeliS trial is a multicentre and multidisciplinary public health project in ten regions of Bavaria, a federal state of Germany, targeting maternal and fetal health. It is designed as a prospective, cluster-randomized, controlled, open intervention trial. We perform a paired cluster-randomization by matching five pairs of regions according to birth figures, sociodemographic and geographic criteria. Randomization is performed within these pairs and each pair is supervised by an expert centre for nutrition run by the Bavarian State Ministry of Food, Agriculture and Forestry.

Adherence to the study protocol is monitored by the Münchner Studienzentrum at the Technical University of Munich. The Münchner Studienzentrum is furthermore in charge of comprehensive data management according to current standards. The study will be conducted in accordance with the declaration of Helsinki as well as with current local regulatory requirements and laws. Written informed consent will be given by all participants. The study protocol was approved by the ethical committee of the Technical University of Munich and registered at the ClinicalTrials.gov Protocol Registration System (NCT01958307).

### Power calculation

The sample size was calculated based on the proportion of women exceeding weight gain recommendations according to the Institute of Medicine (IOM) guidelines and the results of the FeLIPO study which was conducted as a pilot study [44]. In accordance with data from the Bavarian perinatal register, it is expected that at least 40% of the women (BMI > 18.5 kg/m^2^) will exceed the threshold levels of IOM in the control group (Beyerlein A, November 2009, personal communication on the basis of Bavarian perinatal data from 2007). A decrease to 30% in the intervention group by adopting the lifestyle intervention programme is assumed. To detect this difference in proportions with 90% power using a significance level (alpha) of 0.05 and an intraclass correlation coefficient of 0.5% [46], 1900 pregnant women are needed [47]. To compensate for a possible imbalance in the sample sizes between groups as well as a drop-out rate of up to 25% until delivery, 2500 participants are required for the trial and it is planned to recruit these women in both urban and rural regions reflecting the socioeconomic conditions in the Bavarian population.

### Study population

#### Inclusion criteria

Women aged 18 to 43 years with a singleton pregnancy, a pre-pregnancy BMI ≥ 18.5 kg/m^2^ and ≤ 40 kg/m^2^, and sufficient German language skills are eligible for the study. These pregnant women are recruited before the 12^th^ week of gestation and written informed consent is mandatory.

#### Exclusion criteria

Exclusion criteria are multiple pregnancy, high risk pregnancy prohibiting study participation (contraindications to exercise e.g. placenta praevia, persistent bleeding, cervical incompetence etc.), pre-pregnancy diabetes mellitus or early gestational diabetes, uncontrolled chronic diseases (e.g. thyroid dysfunction), psychiatric or psychosomatic diseases, and any other diseases which could interfere with compliance according to the study protocol.

#### Drop-out criteria

Reasons for drop-out are complications during pregnancy as assessed by the trial physician. Women who give birth preterm (delivery before 37^th^ week of gestation) will be excluded from the GWG analysis. These and other reasons for drop-out are carefully documented.

#### Counsellor recruitment and qualification

All practice-based gynaecologists including their medical staff as well as all midwives in the selected regions are contacted via information leaflets and by phone. Participating gynaecologists, medical staff or midwives will act as local health care providers and key communicators by implementing counselling sessions for participating pregnant women in the “real-life” setting of routine care. They have to attend a standardised qualifying seminar to ensure competency in lifestyle counselling and standardisation of the intervention. Seminar and information material for the counsellors as well as for the pregnant women were developed in cooperation with the network “Healthy Start - Young Family Network”, a project of IN FORM, which is funded by the German Federal Ministry of Food and Agriculture [48].

#### Participant recruitment

Before the 12^th^ week of gestation, all pregnant women consecutively presenting in the participating practices are invited by midwives or medical staff to take part in the study. To check inclusion and exclusion criteria and to obtain basic information, participating as well as non-participating women are requested to fill in a short screening questionnaire which includes demographic and anamnestic data relevant to the study. If women fulfill all criteria and are interested to participate, they receive detailed information about the study as well as a form to provide their written informed consent. All these materials were approved by the ethical committee.

### The lifestyle intervention programme

**Figure 1:**
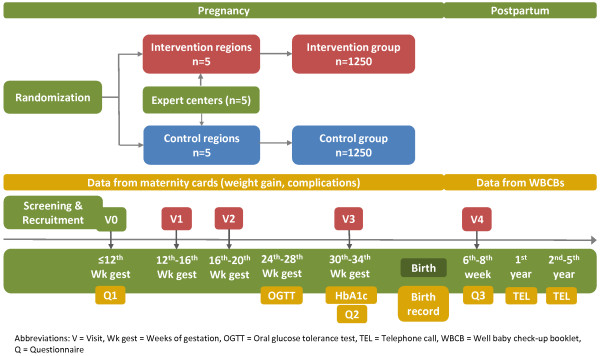
Study scheme of the GeliS study.

The intervention programme consists of four structured and partially individualised counselling sessions emphasizing diet, physical activity and weight monitoring (12^th^-16^th^, 16^th^-20^th^, and 30^th^-34^th^ week of gestation and 6^th^-8^th^ week postpartum). The counselling sessions are exclusively given by specifically trained and certified midwives, gynaecologists or medical staff alongside routine prenatal visits and follow a defined curriculum. Pregnant women receive a pedometer and brochures including examples for adequate exercise and a list of local prenatal physical exercise programmes as well as recommendations for a balanced diet in pregnancy according to the above mentioned recommendations on nutrition in pregnancy [49]. Furthermore, they receive a weight gain chart according to their baseline BMI category for self-monitoring of weight development as proposed by the IOM [16]. Figure 1 shows the study scheme including all visits, examinations and other procedures as well as the data that is taken from the pregnant women/mothers and their newborns.

*Visit 0* (≤ 12^th^ week of gestation) represents the screening examination. After checking inclusion and exclusion criteria and obtaining written informed consent, each participant obtains a folder containing questionnaires (on dietary habits and supplements/physical activity/maternal mental health) as well as a pedometer (Beurer GmbH, Ulm, Germany) with adequate instructions.

*Visit 1* (12^th^-16^th^ week of gestation): Detailed information about healthy diet and physical activity in pregnancy is given in a face-to-face session of 30 to 45 min. duration. The importance of a healthy lifestyle during pregnancy and its determining factors are explained. Pregnant women learn to handle the principles of healthy eating and to understand the risks of alcohol, smoking and foodborne infections during pregnancy. Further advice relates to GWG, weight monitoring and to critical nutrients in pregnancy (folic acid, iodine, and iron). A list of adequate prenatal physical exercise programmes, brochures including recommendations for a balanced diet in pregnancy as well as a chart for adequate weight gain according to baseline BMI category to self-monitor weight development are handed out. The questionnaires distributed in *Visit 0* are collected.

*Visit 2* (16^th^-20^th^ week of gestation, 4 weeks distance between the two visits): Specific and detailed individual counselling targeting dietary habits and physical activity also considering the information from the first set of questionnaires is given. Moreover, women are motivated to participate in a standardised two hour oral glucose tolerance test (OGTT) between the 24^th^ and 28^th^ week of gestation, and an appointment for the OGTT is fixed.

*Visit 3* (30^th^-34^th^ week of gestation): Repetition and consolidation of contents from the previous visits, with an emphasis on weight monitoring. Counsellors provide information on prenatal maternity courses, post natal exercise classes and on specific problems, such as water retention or back complaints. In addition, the importance of breastfeeding is addressed. A venous EDTA blood sample for measurement of glycosylated haemoglobin (HbA1c) is collected. The second set of questionnaires is handed out.

*Visit 4* (6^th^-8^th^ week postpartum): The fourth counselling session includes dietary advice during breastfeeding and information on infant feeding principles. Full breastfeeding is recommended at least up to the age of 4 months. If not returned yet, the second set of questionnaires is collected and the third set of questionnaires (dietary habits and intake of supplements/physical activity/post natal depression/evaluation) is handed out together with a free envelope to return the questionnaires. Maternal weight is measured and documented at each visit.

### Control group

Participants in the control group receive standard prenatal care, including a leaflet about a healthy lifestyle in pregnancy, but no specific individual advice on diet, physical activity or weight gain in pregnancy is given. Data collection and distribution of questionnaires take place within routine prenatal care visits at the same time periods as in the intervention group.

### Study outcomes

#### Primary outcome

The primary outcome parameter is the proportion of participating women with excessive GWG as defined by the Institute of Medicine (IOM) [16].

#### Secondary outcomes

Secondary outcome parameters include:

– incidence of gestational diabetes (24^th^-28^th^ week of gestation, via an oral glucose tolerance test), glycosylated haemoglobin concentration (30^th^-34^th^ week of gestation),

– other pregnancy complications such as preeclampsia,

– anthropometric measures and health status of the newborns (birth weight, height, head circumference, LGA, small for gestational age (SGA), APGAR-Score, pH),

– obstetric complications (mode of delivery, induction of labour, rate of caesarean sections etc.),

– maternal dietary and physical activity habits during pregnancy and after delivery,

– maternal body weight after delivery (6-8 weeks postpartum),

– maternal mental health and post natal depression.

#### Follow-Up

It is planned to add a follow-up observation programme up to the age of 5 years. Follow-up parameters include breastfeeding and infant feeding practices, growth development and health status of the offspring (including weight and height measurement) as well as maternal weight development.

### Outcome measures and data collection

#### Weight measurements, documentation of pregnancy and obstetric complications

GWG is calculated as the difference between maternal weight at the last prenatal visit and weight at the first visit. The self-reported pre-pregnancy weight is documented at the time of recruitment. At every antenatal visit, weight, blood pressure, urine analysis data as well as pregnancy complications are routinely documented in the maternity card. A standard operation procedure is provided to the participating practices detailing weight measurement. In Germany, maternity cards are delivered to every pregnant woman at their first gynaecological visit as soon as pregnancy is confirmed. To retrieve data regarding complications during pregnancy and delivery and infant anthropometrics, practice staff will copy maternity cards and birth records at the first postnatal visit.

#### Questionnaires

#### Screening-questionnaire

All women contacted are asked to complete a short anonymous questionnaire containing demographic and anamnestic data independent of study participation.

#### Dietary assessment

Dietary habits are recorded twice during pregnancy and once after delivery using a validated Food Frequency Questionnaire (FFQ) according to the DEGS-Study (“German Health Interview and Examination Survey for Adults” conducted by the Robert Koch Institute) [50]. Questions on the intake of dietary supplements are added. Women attending the intervention group receive questionnaires at *Visits 0*, *3* and *4*. Women in the control group receive FFQs at the same time points.

#### Physical activity assessment

Physical activity is assessed at three time points in both groups (analogous to FFQs) using the validated “Pregnancy physical activity questionnaire” (PPAQ), modestly adjusted to German habits [51]. In addition, a subgroup of women in the intervention group (n = 250) and in the control group (n = 250) are asked to carry a pedometer for seven consecutive days. Daily steps are documented in a personal diary which is handed out and completed at the same time points as the FFQ and the PPAQ.

#### Maternal mental health

In collaboration with the Mental Health Research Unit of the Helmholtz Zentrum München we have defined a questionnaire with validated elements (WHO-5; PHQ-4; questions from the KORA study - a research platform that is used by national and international research teams [52]). The mental health questionnaire is handed out and collected at the same time points as the FFQ and the PPAQ.

#### Post natal depression

Using the Edinburgh Postnatal Depression Scale (EPDS-Scale) postnatal depression is recorded at 6-8 weeks after delivery. The German version validated by Bergant et al. [53] will be used.

#### Evaluation

To assess the acceptance of the lifestyle programme, all participants in the intervention group obtain an evaluation form at the end of the intervention (*Visit 4*).

#### OGTT and HbA1c

To test for gestational diabetes mellitus (GDM), a standardised two-hour oral glucose tolerance test (OGTT) is offered to all participants between the 24^th^ and 28^th^ week of gestation. Tests are performed by the participating gynaecological practices according to the 2011 guidelines of the German Diabetes Society (DDG) and the German Society of Gynaecology and Obstetrics (DGGG) [54] which is in line with the guidelines of the International Association of the Diabetes and Pregnancy Study Groups (IADPSG) [55]. After an overnight fast, serum glucose level is measured before, one and two hours after intake of 75 grams glucose (Dextro®OGT, Roche, Mannheim). Treatment will be performed at the discretion of the participating gynaecologist. Every participating gynaecological practice will receive information about the DDG/DGGG guidelines for the management of gestational diabetes [54] and is asked to consider these principles as soon as GDM is diagnosed.

To obtain additional information on the presence and severity of diagnosed GDM, EDTA blood is collected for the measurement of the glycosylated haemoglobin concentration between the 30^th^ and 34^th^ week of gestation.

#### Follow-Up – well baby check-up booklet

Follow-up interviews (by phone call or e-mail, upon preference) will be arranged after one, and annually up to 5 years after delivery to record maternal body weight as well as infant anthropometrics. The infant anthropometrics are based on measured data from the well baby check-up booklet provided to all mothers in Germany at birth to promote regular health examinations by the family pediatricians. This is part of the free health check-up programme for infants in the German health care system. The (preschool-) programme comprises standardised health examinations at six time points during the first year of age (immediately after delivery, 3^th^-10^th^ day of age, 4^th^-5^th^ week of age, 3^th^-4^th^ month of age, 6^th^-7^th^ month of age, 10^th^-12^th^ month of age) as well as annually up to the age of five (21^th^-24^th^ month of age, 34^th^-36^th^ month of age, 46^th^-48^th^ month of age, 60^th^-64^th^ month of age). In Bavaria, the programme is currently utilized by approximately 84.5% of all parents [56]. All mothers are explicitly encouraged to participate in this programme. In addition, breastfeeding and infant feeding practices are assessed and documented. All data are pseudonymised before they are transferred to data management at the Münchner Studienzentrum for database entry.

## Discussion

Excessive GWG is well known to be associated with a higher risk of pregnancy and birth complications [15-19,57,58]. Moreover, excessive GWG has also been reported to favour maternal weight retention [23] and to promote childhood obesity [24-33]. According to recent surveys [16,59,60], an increasing number of pregnant women show excessive GWG as defined by the guideline of the Institute of Medicine [16]. Therefore, there is an urgent demand for effective strategies to stop this trend in order to avoid its potential adverse consequences for both mother and child.

We recently published the results of a feasibility study performed in 8 gynaecological practices in the Munich area using a cluster-randomized design. Two sessions of lifestyle counselling were sufficient to achieve a significant reduction in the percentage of women with excessive GWG compared to a control group. There was also less weight retention in the mothers at month 4 as well as a tendency for a lower rate of gestational diabetes and less caesarean sections [44]. These results are in line with a number of other small intervention studies aiming to limit GWG during pregnancy. Most studies showed a moderate reduction in GWG, but were not adequately powered for other outcome variables such as GDM or obstetric complications [40-43,61-64].

The present study is based on this experience, but aims to explore this concept of lifestyle intervention in the routine health care system. In Germany, regular pre- and postnatal care is well established and utilised by almost every pregnant woman. However, the current health care system for pregnant women and their offspring is solely focussing on obstetrical aspects and, after birth, on the functional development of the children. Our concept is to complement this system by a lifestyle programme combining a healthy diet, regular physical activity, and self-monitoring of body weight to avoid excessive weight gain and its potential risks for both mother and child.

The increased risk of long-term obesity in the offspring of mothers with excessive GWG [24,29,30,33,57] is hypothesized to result from a malprogramming process during pregnancy and early postnatal life [3,65]. This concept of fetal programming is attractive, but since the current evidence is mainly based on animal data and retrospective human association studies it has not yet been proven for humans. Therefore, a specific feature of our trial is to look at weight development in the children up to the age of 5 years utilising the unique health check-up system for children in Germany. Thus, this study may also serve to examine the hypothesis that a healthy lifestyle during pregnancy can reduce the risk of early childhood obesity in the offspring.

Pregnancy and lactation is not only a critical period for long-term malprogramming, but can also be considered as a “teachable moment” describing a life situation that is thought to motivate women to adopt a risk-reducing health behaviour [42]. As already seen in our feasibility study, the compliance with a lifestyle programme may be particularly high in this phase. In the interest of the health of their babies, women are more responsive to lifestyle counselling than at any other time. In addition, if the concept of fetal programming turns out to be valid, a temporarily limited lifestyle change may not only gain more acceptance by women, but could have a sustained beneficial effect.

In addition to the long-term follow-up of the infants, another potential strength of the GeliS study is the “real world” setting. The study is performed within the framework of the German healthcare system and represents a true public health approach to this upcoming health challenge. The intervention programme is paid for by the largest regional statutory health insurance and it is planned to implement the lifestyle intervention programme into routine care if a benefit for both mother and child can be demonstrated. An accompanying health economic analysis is planned to study the cost-effectiveness of the intervention.

A weakness of the study may be that there are other potentially relevant perinatal factors which may modify the obesity risk in this early phase of life. In a recent analysis of birth cohort data pre-pregnancy BMI was identified as the most powerful determinant of childhood obesity [66]. However, a recent longitudinal study in Southampton, UK showed that only a small proportion of women followed nutrition and lifestyle recommendations pre-pregnancy, but this proportion increased during pregnancy [67]. Thus, prevention of excessive GWG together with the other recommendations for a healthy lifestyle, e.g. stopping smoking and alcohol consumption, may be the most promising and practicable strategy to address the lifestyle-associated health issues previously discussed.

In summary, the GeliS trial will examine the potential of a strategy implemented to avoid excessive GWG and, thus, to prevent the GWG-associated health risks for both mother and child in the routine health care of pregnant women. If shown to be effective, it is important to note that the collaborating statutory health insurance fund plans to implement the lifestyle counselling into the routine care programme for pregnant women. Then, promotion of a healthy lifestyle including management of weight gain during pregnancy could become an easy and valuable strategy for the primary prevention of adverse consequences of excessive GWG in both women and their children.

## Competing interests

The authors declare that they have no competing interests.

## Authors’ contributions

HH, KR, JK, ER and LK are members of the GeliS study group and contributed to the design of the study. HH and KR developed the study protocol. HH, KR, ER and LK established the lifestyle intervention programme. KU provided advice on power calculation and statistical analyses. JK, KR, and HH wrote the manuscript. All authors read and approved the final manuscript.

## Pre-publication history

The pre-publication history for this paper can be accessed here:

http://www.biomedcentral.com/1471-2393/14/119/prepub
